# Antenna Combining for Interference Limited MIMO Cellular Networks

**DOI:** 10.3390/s20154210

**Published:** 2020-07-29

**Authors:** Tae-Kyoung Kim

**Affiliations:** Department of Electronics, Information and Communication Engineering, Mokpo National University, Muan, Jeonnam 58554, Korea; tk415kim@gmail.com

**Keywords:** multiuser multi-input multi-output (MU-MIMO), zero-forcing beamforming (ZFBF), limited feedback, quantization-based combining, cellular networks

## Abstract

This paper considers a downlink cellular network where multi-antenna base stations (BSs) simultaneously serve their associated multi-antenna users. Each BS is distributed according to a homogeneous Poisson point process and uses zero-forcing beamforming for spatial division multiplexing with partial channel state information (CSI). During downlink transmission, each user combines the multiple antenna outputs and quantizes the CSI to feed back to its associated BS. Specifically, this paper focuses on antenna combining at the receiver. Conventional quantization-based combining (QBC) effectively reduces the quantization error; however, inter-cell interference in the cellular networks degrades the QBC gain. This degradation is analyzed using a spherical-cap approximation of vector quantization (SCVQ). From the SCVQ, the ergodic spectral efficiency and the optimal number of feedback bits are investigated, and it is shown that the QBC degrades the gain of the effective channel. To address this problem, an optimization solution is proposed that selects the antenna combining to maximize the spectral efficiency. The solution is also derived by considering the expected beamforming vectors of other cells. It is demonstrated by simulation that the proposed solution outperforms the conventional methods.

## 1. Introduction

Multi-input multi-output (MIMO) is a promising technology to meet the demand for high speed in wireless communications. Its capacity linearly increases with the number of antennas, which is called multiplexing gain [1,2,3]. In multiuser (MU)-MIMO broadcast channels, this gain can be effectively accomplished using space division multiple access (SDMA) [4,5]. The SDMA scheme allows for simultaneous transmission of independent data streams to each user. Zero-forcing beamforming (ZFBF) achieves near-optimal spectral efficiency with a simple structure [6,7]. However, to achieve this spectral efficiency, the transmitter requires perfect channel state information (CSI) at the receiver which is generally challenging to achieve in practical systems. However, limited feedback overcomes this challenge to give a partial CSI to the transmitter [8,9,10,11]. With limited feedback, the receiver estimates and quantizes the CSI and feeds it back to the transmitter. As only partial CSI is provided then quantization errors will exist which cause the inevitable multiuser interference.

Various studies have investigated methods to achieve spectral efficiency close to perfect CSI [12,13,14,15,16]. Reference [17] shows that in multi-input single-output (MISO) channels, the number of feedback bits should be linearly increased with the signal-to-noise ratio (SNR). This result can be extended to the MIMO channels by using a quantization-based combining (QBC) scheme at the receiver [18]. The QBC scheme effectively minimizes the quantization error by exploiting multiple receive antennas which are linearly combined to form an effective single antenna channel. The effective single antenna channel provides analytic flexibility in limited feedback systems. By using its flexibility, QBC has been widely investigated in various wireless applications [19,20,21,22,23]. Compared to ZFBF, the number of feedback bits is significantly reduced to combine with the block diagonalization beamforming in [21]. In [22], the QBC scheme is applied to orthogonal frequency division multiplexing systems by using subcarrier grouping. In a recent study, and with user cooperation, the QBC scheme was applied to a machine type communication networks [23]. This demonstrated that the QBC scheme is of benefit in various networks; however, its potential gain has not been investigated in actual cellular networks.

To reflect a realistic cellular network, stochastic geometry has been introduced in [24,25,26,27]. In this model, the base station (BS) and user are randomly located by using random point process. In [28], the spectral efficiency was analyzed by exploiting the stochastic geometry on the assumption of perfect CSI. This study was extended to the imperfect CSI scenario by using a homogeneous Poisson point process (PPP) for BS topology [29]. It was shown that the number of feedback bits is a linear function of the number of transmit antennas and of path loss exponent, and is a logarithmic function of the channel coherence time. Recently, the author in [30] analyzed the analytical bounds when the values of channel coherence time are small. In addition, the number of feedback bits involving a noise effect was analyzed in [31]. These results provided meaningful insight in realistic cellular networks; however, a single antenna user was assumed. The user generally has multiple antennas in the mobile and can increase the potential gain by combining the multiple received outputs.

In this paper, antenna combining is proposed for interference limited MIMO cellular networks. In the considered cellular networks, the ergodic spectral efficiency and the optimal number of feedback bits for the QBC method are first analyzed. The analysis is based on the spherical-cap approximation of vector quantization (SCVQ) model. On the assumption of many feedback bits, it is shown that the effective channel gain is reduced by the QBC method although it effectively reduces quantization error. To overcome the reduction in gain, an antenna combining solution is proposed for interference limited cellular networks. The optimization problem is first introduced to select an appropriate antenna combining solution which maximizes the ergodic spectral efficiency. The ergodic spectral efficiency is averaged over the quantization error and beamforming vectors of other cells, and then the optimal solution is provided based on this ergodic spectral efficiency. The analysis shows that the results obtained from the proposed SCVQ model are consistent with the simulation results and that the proposed antenna combining selection outperforms the conventional antenna combining. Our key contributions in this paper are summarized as follows;

- Conventional antenna combining methods are investigated in an interference limited MIMO cellular network. In this network, it is found that the gain of the QBC method is limited because the inter-cell interference is more dominant than the intra-cell multiuser interference induced by a quantization error. Therefore, the QBC method has a lower performance than the maximum-ratio combining (MRC) method despite the small number of feedback bits.
- The QBC method is proposed for interference limited MIMO cellular networks. Conventional analysis framework described in [18,23,32] using random vector quantization (RVQ) is not applicable in interference limited MIMO cellular networks. Thus, the SCVQ model is approximated on the assumption of many feedback bits. From this approximation, it is shown that the QBC method reduces the dimension of the effective single antenna channel to $N_{t}-N_{r}+1$ where the numbers of transmit and receive antennas are $N_{t}$ and $N_{r}$ respectively. Accordingly, the ergodic spectral efficiency and the optimal number of feedback bits for the QBC method are reduced compared to that of the MRC method where the number of feedback bits increases.
- A selective antenna combining solution is proposed to overcome the reduction. The optimization problem is first introduced that enables selection of the antenna combining solution to maximize the ergodic spectral efficiency. Because the inter-cell interference is important in selecting the antenna combining, especially for cellular networks, the inter-cell interference is only averaged over beamforming vectors of other cells and the distance information in the cell interference is conserved. The required number of other cells to measure the inter-cell interference is derived from the simulation.

This remainder of this paper is organized as follows; Section 1 describes the system model and performance metrics; Section 2 explains the antenna combining techniques such as MRC and QBC; Section 3 derives the performance for interference limited MIMO cellular networks; Section 4 presents the proposed antenna combining selection and the simulation results, and Section 5 concludes the paper.

Notations: Matrix $I_{m}$ is an m×m identity matrix. Superscripts ${\left(\cdot \right)}^{H}$ denotes the conjugate transpose. Operators ∥·∥, |·|, E(·), and P(·) denote vector norm, absolute, expectation, and probability, respectively. Functions Γ(·), B(·), and a2F1(a,b,c,z) are the gamma function, beta function, and Gauss-hypergeometric function, respectively. Distributions g(a,b) and β(a,b) denote Gamma distribution with shape *a* and scale *b* and Beta distribution with parameters *a* and *b*, respectively. The sets N, R, and C denote the set of natural numbers, real numbers, and complex numbers, respectively.

## 2. System Model

### 2.1. Signal Model

A downlink cellular network model is considered in this paper. The BSs are randomly located according to a homogeneous PPP Φ of density λ. Each BS with $N_{t}$ transmit antennas has a coverage region characterized by a Voronoi tessellation and serves *K* users distributed in the Vornoi region. Each user with $N_{r}$ receives antennas is independently distributed and its distribution is independent of Φ.

**Figure 1:**
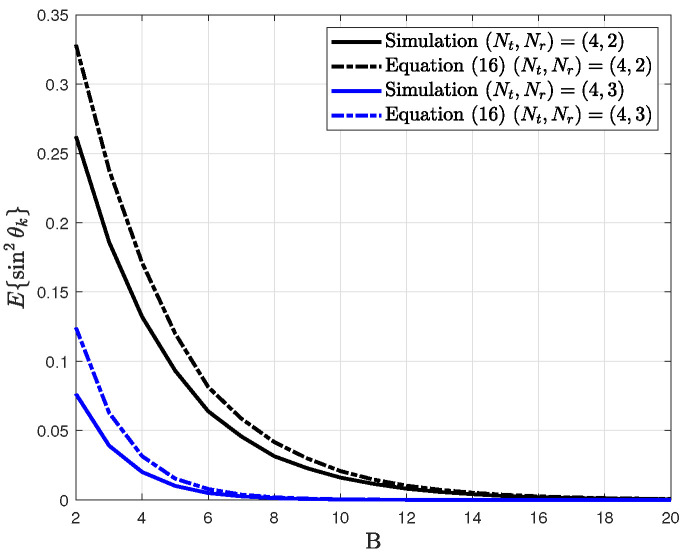
Verification for the distribution of quantization error according to number of feedback bits.

In this network model, the spectral efficiency is investigated for an arbitrary user. By Slivnyak–Mecke’s theorem [33], the user is assumed to be located at the origin without loss of generality The user located at the origin is denoted by the index *k* and the serving BS of user *k* by $b_{k}$. When the distance from the BS *i* and the user *k* is denoted by $d_{i}\in R^{2}$, the homogeneous PPP Φ is defined as $\left\{d_{i},i\in N\right\}$. In Figure 1, a PPP-based cellular network model is illustrated with $\lambda =2\times 10^{-3}$. From the described cellular network model, the received signal of the user *k* is expressed by

(1) $$\begin{array}{cc} y_{k} & =∥d_{b_{k}}{∥}^{-\frac{\alpha }{2}}H_{k,b_{k}}^{H}V_{b_{k}}s_{b_{k}}+\sum_{i=1,i\neq b_{k}}^{\infty }{∥d_{i}∥}^{-\frac{\alpha }{2}}H_{k,i}^{H}V_{i}s_{i}+z_{k}, \end{array}$$

where $H_{k,i}\in C^{N_{t}\times N_{r}}$ is the channel matrix of user from BS *i* to user *k*. $V_{i}=\left[v_{1,i},\ldots ,v_{K,i}\right]\in C^{N_{t}\times K}$ is the beamforming matrix of BS *i* with $∥v_{k,i}∥=1$. $s_{i}\in C^{K\times 1}$ is the transmitted signal vector at BS *i*. $z_{k}\in C^{N_{r}\times 1}$ is complex additive white Gaussian noise which has zero mean and a covariance matrix that is the identity matrix. It is assumed that the path loss exponent is α>2 and that an equal amount of power is allocated to all the users such that $E\left\{s_{i}s_{i}^{H}\right\}=\frac{P}{N_{t}}I_{K}$. *P* is the total transmit power.

For simplicity, $b_{k}$ is omitted in Equation (1). Accordingly, $∥d_{b_{k}}∥$, $h_{k,b_{k}}$, $V_{b_{k}}$, and $s_{b_{k}}$ are replaced with ∥d∥, $h_{k}$, V, and s, respectively. Then, the equation in Equation (1) is re-expressed as

(2) $$\begin{array}{cc} y_{k} & ={∥d∥}^{-\frac{\alpha }{2}}H_{k}^{H}v_{k}s_{k}+\sum_{k^{\prime }=1,k^{\prime }\neq k}^{K}{∥d∥}^{-\frac{\alpha }{2}}H_{k}^{H}v_{k^{\prime }}s_{k^{\prime }}+\sum_{i=1,i\neq b_{k}}^{\infty }{∥d_{i}∥}^{-\frac{\alpha }{2}}H_{k,i}^{H}V_{i}s_{i}+z_{k}. \end{array}$$

As in [18], it is assumed that single data stream for each user and linear antenna combining for the received signal $y_{k}$. Then, the received signal (scalar) after antenna combining can be rewritten as

(3) $$\begin{array}{cc} \gamma_{k}^{H}y_{k} & ={∥d∥}^{-\frac{\alpha }{2}}{\left(h_{k}^{e}\right)}^{H}v_{k}s_{k}+\sum_{k^{\prime }=1,k^{\prime }\neq k}^{K}{∥d∥}^{-\frac{\alpha }{2}}{\left(h_{k}^{e}\right)}^{H}v_{k^{\prime }}s_{k^{\prime }}+\sum_{i=1,i\neq b_{k}}^{\infty }{∥d_{i}∥}^{-\frac{\alpha }{2}}{\left(h_{k,i}^{e}\right)}^{H}V_{i}s_{i}+z_{k}, \end{array}$$

where $\gamma_{k}\in C^{N_{r}\times 1}$ is unit-norm antenna combiner. $h_{k}^{e}=H_{k}\gamma_{k}\in C^{N_{t}\times 1}$ and $h_{k,i}^{e}=H_{k,i}\gamma_{k}\in C^{N_{t}\times 1}$ are the effective channels from BS $b_{k}$ and from BS *i* to user *k*, respectively. $z_{k}=\gamma_{k}^{H}z_{k}$ is a complex Gaussian random variable with zero mean and unit variance.

### 2.2. Quantization-Based Combining

The QBC method finds the optimal effective channel to minimize a quantization error. When $Q_{k}\in C^{N_{t}\times N_{r}}$ is the orthonormal basis of span($H_{k}$), the receiver finds the quantization vector that is close to span($H_{k}$) from the codebook $C_{k}$. Then, the quantized CDI is obtained as

(4) $$\begin{array}{cc} {\hat{h}}_{k}^{e} & =\underset{c\in C_{k}}{\mathrm{argmax}}{∥Q_{k}c∥}^{2}. \end{array}$$

The effective channel of Equation (4) can be maximized by projecting ${\hat{h}}_{k}^{e}$ onto span($H_{k}$) such as

(5) $$\begin{array}{cc} s_{k}^{p} & =\frac{Q_{k}Q_{k}^{H}{\hat{h}}_{k}^{e}}{∥Q_{k}Q_{k}^{H}{\hat{h}}_{k}^{e}∥}, \end{array}$$

where $s_{k}^{p}$ is the direction to minimize the quantization error in the channel space of $H_{k}$. The normalized combining vector $\gamma_{k}^{q}$ is calculated as follows

(6) $$\begin{array}{cc} \gamma_{k}^{q} & =\frac{{\left({\hat{H}}^{H}\hat{H}\right)}^{-1}{\hat{H}}^{H}s_{k}^{p}}{∥{\left({\hat{H}}^{H}\hat{H}\right)}^{-1}{\hat{H}}^{H}s_{k}^{p}∥}, \end{array}$$

where $N_{t}>N_{r}$ is assumed because the QBC method does not properly work when $N_{t}\leq N_{r}$ [18].

### 2.3. Finite Rate Feedback Model

In finite rate feedback model, each user quantizes the effective channel direction information (CDI) by using the codebook. A random vector quantization (RVQ) is used to generate a predefined codebook $C_{k}=\left\{c_{k,1},\ldots ,c_{k,2^{B}}\right\}$. This codebook is known to the BS and its associated user. Each codeword in the codebook is a $N_{t}$-dimensional unit-norm vector $∥c_{k,i}∥=1$ for $i=\{1,\ldots ,2^{B}\}$ where *B* is the number of feedback bits. The distance between the codeword $c_{k,i}$ and the normalized CDI of ${\tilde{h}}_{k}^{e}=\frac{h_{k}^{e}}{∥h_{k}^{e}∥}$ is measured by the inner product. Then, the quantized CDI is chosen among the inner products as

(7) $$\begin{array}{c} {\hat{h}}_{k}^{e}=\underset{c\in C_{k}}{\mathrm{argmax}}\left|{\left({\tilde{h}}_{k}^{e}\right)}^{H}c\right|. \end{array}$$

Because the codebook $C_{k}$ is shared between the BS and its associated user, the quantized index of ${\hat{h}}_{k}^{e}$ is fed back to the BS. In this paper, perfect channel information $h_{k}^{e}$ is assumed at the receiver to enable the focus to be on the antenna combining effect.

Let the difference between $\left({\tilde{h}}_{k}^{e}\right)$ and ${\hat{h}}_{k}^{e}$ be the quantization error. Then, the quantization error can be characterized by $\theta_{k}$ as

(8) $$\begin{array}{cc} \cos^{2}\theta_{k} & ={\left({\tilde{h}}_{k}^{e}\right)}^{H}{\hat{h}}_{k}^{e}, \end{array}$$

where $\theta_{k}\in \left[0,\frac{\pi }{2}\right]$. The normalized effective channel ${\tilde{h}}_{k}^{e}$ can be decomposed by $\theta_{k}$ as

(9) $$\begin{array}{cc} {\tilde{h}}_{k}^{e} & =\cos\theta_{k}{\hat{h}}_{k}^{e}+\sin\theta_{k}g_{k}, \end{array}$$

where $g_{k}$ is a unit vector that is isotropically distributed in the null space of ${\hat{h}}_{k}^{e}$.

After the BS acquires the quantized CDI, it implements a beamforming matrix $v_{j}$. As a SDMA scheme, ZFBF is considered because it provides a near-optimal performance despite its simple structure. The ZFBF is designed to satisfy

(10) $$\begin{array}{c} {\hat{h}}_{k}^{e}v_{k^{\prime }}=0,\;\forall k^{\prime }\neq k \end{array}$$

where $v_{k}$ is a unit-norm vector $∥v_{k}∥=1$.

### 2.4. Performance Metric

From Equation (3), the received signal-to-interference-plus-noise ratio (SINR) of user *k* is defined as

(11) $$\begin{array}{cc} {\mathrm{SIN}R}_{k}\; & =\frac{|{\left(h_{k}^{e}\right)}^{H}v_{k}{|}^{2}}{\sum_{k^{\prime }=1,k^{\prime }\neq k}^{K}|{\left(h_{k}^{e}\right)}^{H}v_{k^{\prime }}{|}^{2}+{∥d∥}^{\alpha }\sum_{i=1,i\neq b_{k}}^{\infty }∥d_{i}{∥}^{-\alpha }∥{\left(h_{k,i}^{e}\right)}^{H}V_{i}{∥}^{2}+{∥d∥}^{\alpha }\frac{K}{P}}=\frac{I_{S}}{I_{U}+I_{C}+I_{N}}, \end{array}$$

where the signal power $I_{S}$, the intra-cell multiuser interference $I_{U}$, the inter-cell interference $I_{C}$, and the noise $I_{N}$ are respectively defined as

(12) $$\begin{array}{cc} I_{S} & =|{\left(h_{k}^{e}\right)}^{H}v_{k}{|}^{2},\\ I_{U} & =\sum_{k^{\prime }=1,k^{\prime }\neq k}^{K}{\left|{\left(h_{k}^{e}\right)}^{H}v_{k^{\prime }}\right|}^{2},\\ I_{C} & ={∥d∥}^{\alpha }\sum_{i=1,i\neq b_{k}}^{\infty }∥d_{i}{∥}^{-\alpha }{∥{\left(h_{k,i}^{e}\right)}^{H}V_{i}∥}^{2},\\ I_{N} & ={∥d∥}^{\alpha }\frac{K}{P}. \end{array}$$

Please note that the interference in Equation (12) is evaluated by considering all possible interfering BSs while a finite number of interfering BSs with λA is used in simulation. The ergodic (downlink) spectral efficiency per user *k* is obtained as

(13) $$\begin{array}{cc} R_{k} & =E[\log_{2}\left(1+{\mathrm{SIN}R}_{k}\right)]. \end{array}$$

The downlink spectral efficiency can increase as the number of feedback bits increases. However, a large number of feedback bits also increases the uplink resource to convey the CDI bits. To evaluate the overall spectral efficiency, the net spectral efficiency [29] is considered as

(14) $$\begin{array}{cc} R_{\mathrm{net},k} & =R_{k}-\frac{B}{T_{c}}, \end{array}$$

where $T_{c}$ is the channel coherence time. During the channel coherence time, all symbols including downlink and uplink experience the same channel fading. Thus, the net spectral efficiency multiplied by $T_{c}$ represents the practical downlink spectral efficiency by subtracting the number of feedback bits for the uplink. Throughout the paper, the net spectral efficiency is used in Equation (14) as a practical performance metric.

## 3. Spherical-Cap Approximation of Vector Quantization-Based Analysis

The QBC performance is investigated by adopting the SCVQ model which is a well-known method to analyze the performance in limited feedback systems. It provides an analytical flexibility by using a quantization cell approximation [7,34]. This assumption produces the performance upper bound however, the performance gap becomes small when *B* is large.

In SCVQ model, each quantization cell is assumed to be a Voronoi region of a spherical cap with the area of $2^{-B}$. Thus, the irregular shape of the Voronoi quantization regions with $R_{i}=\{{\tilde{h}}_{k}^{e}:|{\left({\tilde{h}}_{k}^{e}\right)}^{H}c_{i}{|}^{2}\geq |{\left({\tilde{h}}_{k}^{e}\right)}^{H}c_{j}{|}^{2},\forall j\neq i\}$ is approximated as the regular shape with $R_{i}=\{{\tilde{h}}_{k}^{e}:|{\left({\tilde{h}}_{k}^{e}\right)}^{H}c_{i}{|}^{2}\geq 1-\delta \}$ where δ should be chosen to provide $P\left\{R_{i}\right\}=2^{-B}$. From this model, the cumulative distribution function (CDF) of the quantization error for the effective channel follows Lemma 1.

**Lemma** **1.** *For a large B, the CDF of quantization error is given by*

(15) $$\begin{array}{cc} F_{\sin^{2}\theta_{k}}\left(x\right) & \approx \left\{\begin{array}{cc} 2^{B}x^{N_{t}-N_{r}} & ,0\leq x\leq \delta ,\\ 1 & ,\delta \leq x, \end{array}\right. \end{array}$$

*where $\delta =2^{-\frac{B}{N_{t}-N_{r}}}$.*

**Proof.**  See Appendix A for the proof of Lemma 1. □

The derived CDF is different from that in [18] which is given by $F_{\sin^{2}\theta_{k}}\left(x\right)\approx 2^{B}\left(\begin{array}{c} N_{t}-1\\ N_{r}-1 \end{array}\right)x^{N_{t}-N_{r}}$ for 0≤x≤δ where $\delta ={\left(\begin{array}{c} N_{t}-1\\ N_{r}-1 \end{array}\right)}^{-\frac{1}{N_{t}-N_{r}}}2^{-\frac{B}{N_{t}-N_{r}}}$. The CDF in [18] is obtained from the RVQ model by dropping the (1−x) terms in (A3). Accordingly, its CDF and the CDF in Lemma 1 are derived on the same assumption of many feedback bits *B*. However, compared to the CDF derived from the RVQ model, the proposed CDF is more useful to analyze the limited feedback systems. This is because the quantization cell approximation provides an analytical tractability to characterize the performance of the limited feedback systems rather than designing using the explicit codebook.

From the CDF in Lemma 1, the distribution of quantization error is expressed as

(16) $$\begin{array}{cc} f_{\sin^{2}\theta_{k}} & =\delta \cdot \beta (N_{t}-N_{r},1). \end{array}$$

The distribution of quantization error in [29] where each user has a single antenna is given by $\delta \cdot \beta (N_{t}-1,1)$. Thus, the distribution of quantization error is interpreted such that the number of transmit antennas of the effective channel is $N_{t}-N_{r}+1$.

**Figure 2:**
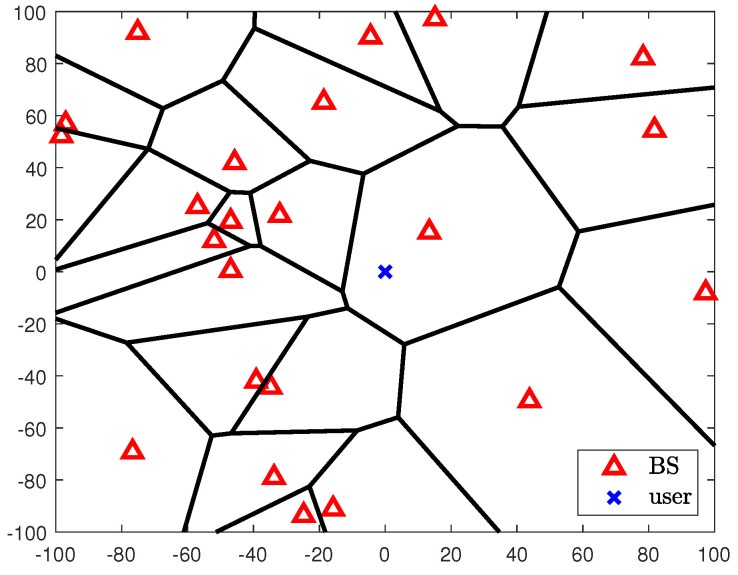
Example of cellular network model by using a homogeneous PPP with $\lambda =2\times 10^{-3}$ in the area of 200×200.

In Figure 2, the distribution of quantization error is compared for the simulation result and analysis result in Equation (16).

It is shown that the simulation result is lower than the analysis result for all cases. The analysis result approaches the simulation result as the number of feedback bits *B* increases. Thus, for a large number of B, the QBC is considered to reduce the number of transmit antennas of the effective channel as $N_{t}-N_{r}+1$. From the distribution in Equation (16), the ergodic spectral efficiency is derived as

**Corollary** **1.** (17) $$\begin{array}{cc} R_{k}\; & \geq \log_{2}\left(1+\frac{e^{\psi (1)}}{\left(K-1\right)2^{-\frac{B}{N_{t}-N_{r}}}+\frac{2K}{\alpha -2}+\frac{K}{P}\frac{\Gamma (1+\frac{\alpha }{2})}{{\left(\lambda \pi \right)}^{\frac{\alpha }{2}}}}\right), \end{array}$$

*where ψ(·) is the digamma function defined as*

(18) $$\begin{array}{cc} \psi (x) & =\int_{0}^{\infty }\left(\frac{e^{-t}}{t}-\frac{e^{-xt}}{1-e^{-t}}\right)dt, \end{array}$$

**Proof.**  By using the lemma 2 in [35], the lower bound of the ergodic spectral efficiency is

(19) $$\begin{array}{cc} E[\log_{2}\left(1+{\mathrm{SIN}R}_{k}\right)]\; & \geq \log_{2}\left(1+\frac{e^{E\{\ln I_{S}\}}}{E\{I_{U}+I_{C}+I_{N}\}}\right), \end{array}$$

where $E\left\{\ln I_{S}\right\}=\psi \left(1\right)$ in [29]. $∥h_{k}{∥}^{2}\sin^{2}\theta_{k}\beta \left(1,N_{t}-2\right)$ follows a distribution of g(1,δ). Thus, $E\left\{I_{U}\right\}=\left(K-1\right)E\left\{g\left(1,\delta \right)\right\}=\left(K-1\right)\delta =\left(K-1\right)2^{-\frac{B}{N_{t}-N_{r}}}$. $∥h_{k,i}^{H}V_{i}{∥}^{2}$ has a distribution of g(K,1) in [31] so that $E\left\{I_{C}\right\}={E\{∥d∥}^{\alpha }\sum_{i=1,i\neq b_{k}}^{\infty }∥d_{i}{∥}^{-\alpha }g\left(K,1\right){\}=KE\{∥d∥}^{\alpha }\sum_{i=1,i\neq b_{k}}^{\infty }∥d_{i}{∥}^{-\alpha }\}=\frac{2K}{\alpha -2}$. and $E\left\{I_{N}\right\}=\frac{K}{P}\int_{0}^{\infty }2\lambda \pi r^{1+\alpha }e^{-\lambda \pi r^{2}}dr=\frac{K}{P}\frac{\Gamma (1+\frac{\alpha }{2})}{{\left(\lambda \pi \right)}^{\frac{\alpha }{2}}}$ where the distribution of ∥d∥ is given in [29]. The proof is completed by combining these results. □

Because the number of feedback bits *B* increases, the feedback period to convey the CDI also increases. Accordingly, the ergodic net spectral efficiency in Equation (14) decreases. Thus, finding the optimal number of feedback bits is an important problem in interference limited cellular networks. This optimal number of feedback bits with respect to channel coherence time $T_{c}$ is derived as

**Corollary** **2.** (20) $$\begin{array}{cc} B_{opt} & \approx \left(N_{t}-N_{r}\right)\frac{\alpha }{2}\log_{2}\left(\rho \left(K\right)T_{c}\phi \left(P,K\right)\right), \end{array}$$

*where ρ(K) and the improper integral ϕ(P,K) are defined as*

(21) $$\begin{array}{cc} \rho (K) & =\frac{\pi \lambda \left(K-1\right)B\left(1-\frac{2}{\alpha },K-1+\frac{2}{\alpha }\right)}{N_{t}-N_{r}},\;\phi \left(P,K\right)=\int_{0}^{\infty }e^{-\frac{K}{P}y^{\frac{\alpha }{2}}-\pi \lambda h\left(K\right)y}dy, \end{array}$$

*where h(K) is given by*

(22) h(K)=2α−2∑l=1Ka2F1(l,1−2α,2−2α,−1)+1−2αK+2a2F1(K,K+2α,K+1+2α,−1).

**Proof.**  For the proof, see Theorem 2 in [31]. □

As the number of receive antennas increases, the quantization error can be much reduced by using the QBC. Due to its quantization error reduction, the effective channel decreases as $N_{t}-N_{r}+1$ in Equation (16). As a result, the effective channel gain is degraded for many *B*. This degradation is undesirable in interference limited cellular networks especially when the inter-cell interference is larger than the intra-cell multiuser interference. In addition, the optimal number of feedback bits decreases as the number of receive antennas increases as in Corollary 2. Critically, $B_{opt}$ approaches zero as the number of receive antennas is close to the number of transmit antennas. Thus, the net spectral efficiency is also difficult to improve by increasing the number of feedback bits.

This degradation is investigated by comparison with the MRC method. The MRC method provides a large channel gain by selecting the dominant subspace of the channel. To obtain the maximum gain in a given channel, it exploits the first column vector that corresponds the largest singular value after singular value decomposition (SVD). Let SVD be $H_{k}=U_{k}\Lambda_{k}S_{k}$ where the diagonal components of $\Lambda_{k}$ are sorted in descending order. Then, the antenna combining vector can be obtained as $\gamma_{k}^{m}=s_{1,k}$ where $S_{k}=\left[s_{1,k},\ldots ,s_{N_{r},k}\right]$.

**Figure 3:**
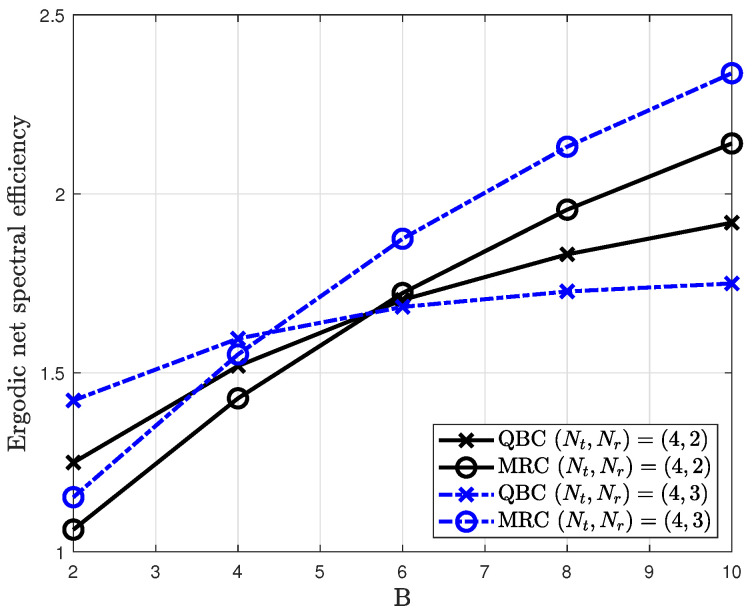
Ergodic net spectral efficiency for different antenna combining methods in the interference limited cellular networks.

Figure 3 shows the ergodic net spectral efficiencies of MRC and QBC according to number of feedback bits. For simulation, 2 and 3 users are considered in the 4×2 and 4×3 MIMO systems at 0 dB, respectively. The rest of the parameters for the cellular network model are explained in Section 4. In the case of a small number of feedback bits, the QBC method outperforms the MRC method due to the quantization error being dominant in this region. However, as the number of feedback bits increases, the ergodic net spectral efficiency of the MRC method is better than that of the QBC method. This is due to the quantization error being negligible for a large number of feedback bits. In addition, it is found that in the 4×3 MIMO systems, the MRC method has a better performance than the QBC method despite the small number of feedback bits (B≥6). This is because the inter-cell interference is larger than the multiuser interference due to the inherent quantization error. In this region, the maximization of the effective channel gain has a more dominant effect than the minimization of quantization error. As the cellular networks are denser, this observation is more critical despite the limited feedback systems.

## 4. Proposed Antenna Combining Method

In this section, an antenna combining selection problem is first considered. The proposed problem relates to the maximization of the spectral efficiency for each user. Hence, to calculate the spectral efficiency accurately, the user should know information about other cells such as distance and beamforming vector. This information is impractical to share so the exact spectral efficiency cannot be derived. Therefore, to relax this constraint, the expected value averaged over the beamforming vectors of other cells is used instead. From this value, a selective antenna combining method is proposed.

### 4.1. Problem Formulation

Although the QBC method effectively minimizes the channel quantization error at the receiver, it also reduces the dimension of the effective channel as investigated in Section 4. As a result, the spectral efficiency is vulnerable to inter-cell interference. For large inter-cell interference, antenna combining to increase the channel gain may be preferable. As a result of this, the optimization problem is formulated to find the optimal combining vector which maximizes the spectral efficiency.

Let be $\Omega_{k}=\left\{m,q\right\}$ to denote an antenna combining set whose elements represents the MRC and QBC methods, respectively. Then, the proposed optimization problem is given by

(23) $$\begin{array}{cc} \underset{i}{\mathrm{argmax}} & \;\log_{2}\left(1+{\mathrm{SIN}R}_{k}\left(\gamma_{k}^{i}\right)\right),\\ \; & \mathrm{subject}\;\mathrm{to}\;i\in \Omega_{k}, \end{array}$$

where the SINR is a function of antenna combining $\gamma_{k}$.

### 4.2. Proposed Algorithm

To solve the optimization problem in Equation (23), each user should calculate an accurate SINR based on the distances and beamforming vectors of other cells. However, they have not been determined at the receiver, so an accurate SINR is difficult to obtain. To address this difficulty, the ergodic spectral efficiency is used as

(24) $$\begin{array}{cc} \underset{i}{\mathrm{argmax}} & \;E\{\log_{2}\left(1+{\mathrm{SIN}R}_{k}\left(\gamma_{k}^{i}\right)\right)\},\\ \; & \mathrm{subject}\;\mathrm{to}\;i\in \Omega_{k}. \end{array}$$

Because the problem to maximize the ergodic spectral efficiency is equivalent to that maximizes the expectation of SINR, the optimization problem is further expressed as

(25) $$\begin{array}{cc} \underset{i}{\mathrm{argmax}} & \;\log_{2}\left(1+E\{{\mathrm{SIN}R}_{k}\left(\gamma_{k}^{i}\right)\}\right),\\ \; & \mathrm{subject}\;\mathrm{to}\;i\in \Omega_{k}. \end{array}$$

When an antenna combining γ is used, the expected SINR in Equation (25) is calculated as

(26) $$\begin{array}{cc} \; & E\{{\mathrm{SIN}R}_{k}\left(\gamma_{k}\right)\}\\ \; & =E\left\{\frac{∥h_{k}^{e}{∥}^{2}{\left|{\left({\tilde{h}}_{k}^{e}\right)}^{H}v_{k}\right|}^{2}}{∥h_{k}^{e}{∥}^{2}\sin^{2}\theta_{k}\sum_{k^{\prime }\neq k}^{K}{\left|g_{k}^{H}v_{k^{\prime }}\right|}^{2}+I_{C}+I_{N}}\right\}\overset{\left(a\right)}{=}E\left\{\frac{∥h_{k}^{e}{∥}^{2}\beta \left(1,N_{t}-1\right)}{∥h_{k}^{e}{∥}^{2}\sin^{2}\theta_{k}\sum_{k^{\prime }=1,k^{\prime }\neq k}^{K}\beta \left(1,N_{t}-2\right)+I_{C}+I_{N}}\right\}\\ \; & \overset{\left(b\right)}{\geq }\frac{E\{∥h_{k}^{e}{∥}^{2}\beta \left(1,N_{t}-1\right)\}}{E\{∥h_{k}^{e}{∥}^{2}\sin^{2}\theta_{k}\sum_{k^{\prime }=1,k^{\prime }\neq k}^{K}\beta \left(1,N_{t}-2\right)+I_{C}+I_{N}\}}=\frac{∥h_{k}^{e}{∥}^{2}\frac{1}{N_{t}}}{∥h_{k}^{e}{∥}^{2}\sin^{2}\theta_{k}\frac{K-1}{N_{t}-1}+E\left\{I_{C}\right\}+E\left\{I_{N}\right\}}. \end{array}$$

In (a), ${\tilde{h}}_{k}^{e}$ and $v_{k}$ are independent and isotropically distributed in $C^{N_{t}\times 1}$. In addition, $v_{k^{\prime }}$ is isotropic within the hyperplane and is independent of $g_{k}$ [7]. Jensen’s inequality is used in (b).

The remaining task is to calculate $E\{I_{C}\}$ and $E\{I_{N}\}$. These can be averaged over $∥d_{i}∥$ and $V_{i}$ as $\frac{2K}{\alpha -2}$ and $\frac{\Gamma (1+\frac{\alpha }{2})}{{\left(\lambda \pi \right)}^{\frac{\alpha }{2}}}$ in Equation (19), respectively. However, this expectation degrades the spectral efficiency when it is applied to the optimization problem. This is because the inter-cell interference value is not appropriately compared with the quantization error in the SINR calculation. In the inter-cell interference, the beamforming vectors of other cells is difficult to obtain in practice. Relatively, the distance information can be obtained from the dedicated reference signal from other cells. Thus, the inter-cell interference is averaged over the beamforming vectors $V_{i}$.

The distance information $∥d_{i}∥$ of other cells in the inter-cell interference is fixed because it is usually unchanged for slow mobile users. However, the infinity sum in the inter-cell interference makes it difficult to calculate. To resolve this problem, the BSs close to the user are considered. For this, the design parameter of $N^{*}$ is introduced in the inter-cell interference where the BS is sorted in ascending order by the distance from user *k*. Then, the inter-cell interference term is approximated as

(27) $$\begin{array}{cc} E\{I_{C}\} & ={E\{∥d∥}^{\alpha }\sum_{i=1,i\neq b_{k}}^{\infty }∥d_{i}{∥}^{-\alpha }∥{\left(h_{k,i}^{e}\right)}^{H}V_{i}{∥}^{2}{\}\approx ∥d∥}^{\alpha }\sum_{i=1,i\neq b_{k}}^{N^{*}}{∥d_{i}∥}^{-\alpha }K, \end{array}$$

where $∥{\left(h_{k,i}^{e}\right)}^{H}V_{i}{∥}^{2}$ follows a distribution of g(K,1). Similarly, it is given by

(28) $$\begin{array}{cc} E\{I_{N}\} & ={∥d∥}^{\alpha }\frac{K}{P} \end{array}$$

By applying these results, the expectation of SINR for given $\gamma_{k}$ is derived as

(29) $$\begin{array}{cc} E\{{\mathrm{SIN}R}_{k}\left(\gamma_{k}\right)\} & =\frac{\frac{1}{N_{t}}{∥h_{k}^{e}∥}^{2}}{∥h_{k}^{e}{∥}^{2}\sin^{2}\theta_{k}\frac{K-1}{N_{t}-1}+{∥d∥}^{\alpha }\sum_{i=1,i\neq b_{k}}^{N^{*}}∥d_{i}{∥}^{-\alpha }K+{∥d∥}^{\alpha }\frac{K}{P}}, \end{array}$$

The optimization problem in Equation (25) has a discrete set of constraint. The optimal solution can be obtained by exhaustively searching in the discrete set. The exhaustive search algorithm is summarized in Algorithm 1. As shown in Algorithm 1, the proposed algorithm first calculates the antenna combining for the candidate. Then, the expected SINR is calculated based on the antennas combining. By using the comparator with the expected SINR, the optimal solution of Equation (26) is finally obtained.
**Algorithm 1:** Proposed algorithm1 **Initialization:**2 Obtain the channel information $H_{k}$3 **for**$i=1:|\Omega_{k}|$**do**4  ($j\leftarrow \Omega_{k}\left(i\right)$5  Calculate the antenna combining $\gamma_{k}^{j}$6  Compute the effective channel $h_{k}^{e}\leftarrow H_{k}\gamma_{k}^{j}$7  Obtain the distance d and $d_{i}$, $i=1,\ldots ,N^{*}$8  Calculate the expected SINR $E\{{\mathrm{SIN}R}_{k}\left(\gamma_{k}^{j}\right)\}$ in [28]9 **end**10 $\hat{i}={\mathrm{argmax}}_{i\in \Omega_{k}}\log_{2}\left(1+E\left\{{\mathrm{SIN}R}_{k}\left(\gamma_{k}^{j}\right)\right\}\right)$11 Determine the antenna combining vector $\gamma_{k}^{\hat{i}}$.12 Select the codebook index from the effective channel $h_{k}^{e}\left(\gamma_{k}^{\hat{i}}\right)$13 Obtain the quantized CDI ${\hat{h}}_{k}^{e}\left(\gamma_{k}^{\hat{i}}\right)$ and its index $c_{k}^{\hat{i}}$13 Feedback the quantized index $c_{k}^{\hat{i}}$ to the BS.

## 5. Simulation Results

In this section, the proposed SCVQ model is first verified in an interference limited cellular network. Then, the ergodic net spectral efficiency and the behavior of the optimal number of feedback bits of the proposed system are investigated. Finally, the proposed antenna combining selection is compared with the existing methods. For BS geometry, a homogeneous PPP with density $\lambda =10^{-5}/\pi$ is considered with a pathloss exponent α=4. The number of BS is generated by Poisson distribution of λA where *A* is the area of $10^{8}$. The received SNR and the coherence time are respectively set to $P_{r}=10$ dB and $T_{c}=200$ unless they are specified. The received SNR is defined as

(30) $$\begin{array}{cc} P_{r} & =\frac{P}{K}\frac{1}{E\{∥d{∥}^{2}\}}=\frac{P}{K}\frac{{\left(\lambda \pi \right)}^{\frac{\alpha }{2}}}{\Gamma (1+\frac{\alpha }{2})}. \end{array}$$

The averaged value in Equation (30) is used because the received SNR is different from the BS geometry.

**Figure 4:**
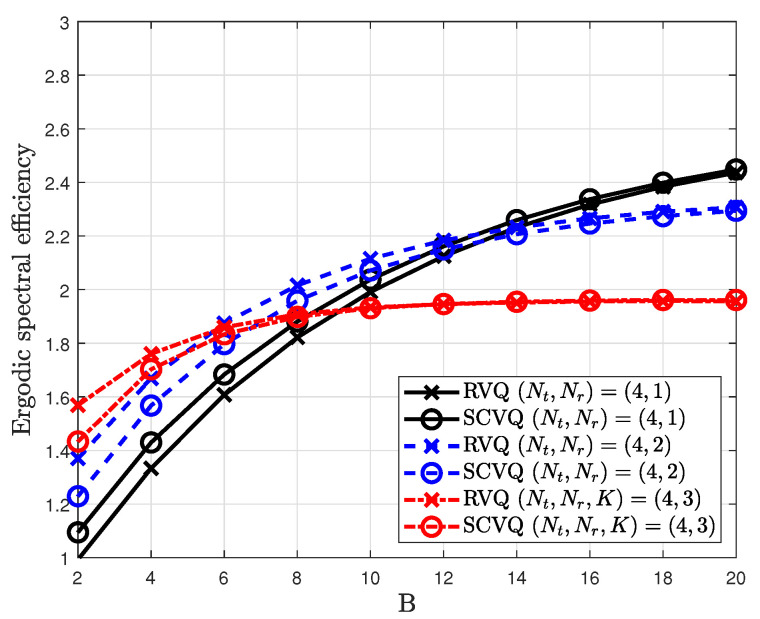
Verification of analysis with the SCVQ model and simulation with the RVQ model.

In Figure 4, the simulation and analysis results in Equation (15) are compared for different MIMO systems.

**Figure 5:**
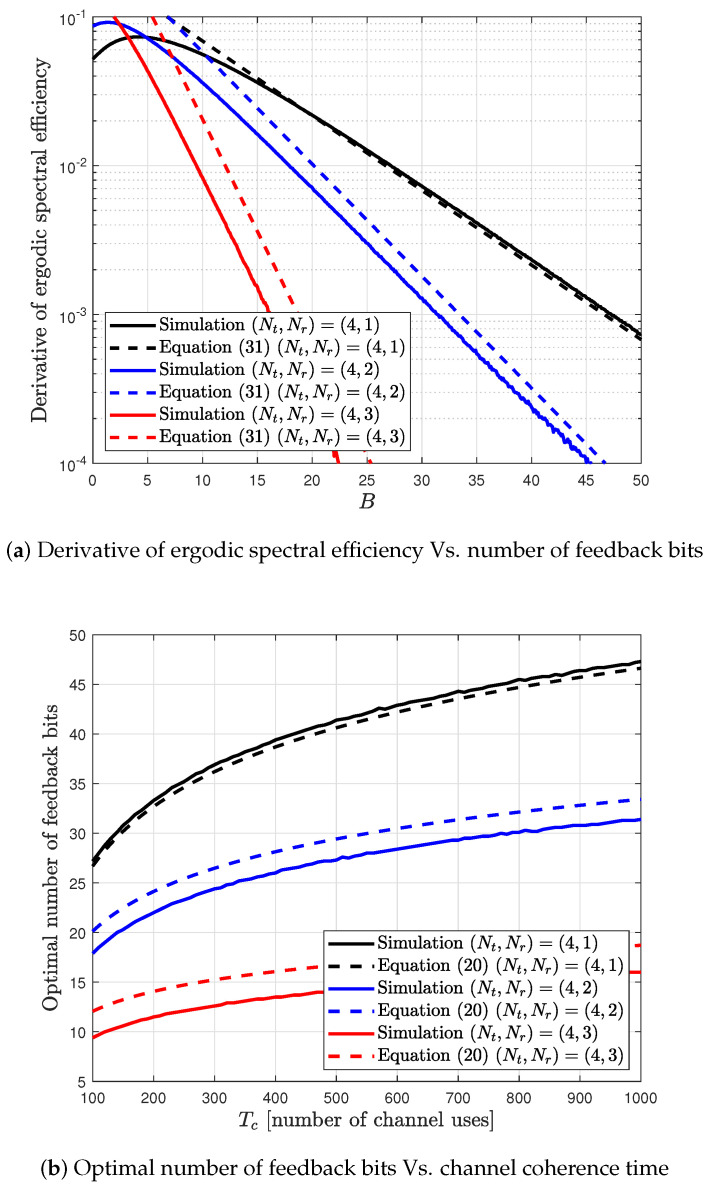
Verification of the optimal number of feedback bits for analysis result.

For analysis, the proposed SCVQ model is assumed to generate a quantized channel as described in Section 1. With the proposed SCVQ model, the quantized CDI ${\hat{h}}_{k}$ can be generated for each B∈R. From the generated CDI ${\hat{h}}_{k}$, the analysis result is obtained. The proposed SCVQ model is close to its actual SCVQ model as the number of feedback bits *B* increases. Thus, the analysis result approaches the simulation result for many feedback bits. Figure 5 shows that the verification of the analysis result derived from Equation (20).

The derivative of ergodic spectral efficiency is simulated by using the proposed SCVQ model. It is given in [31] by

(31) $$\begin{array}{cc} \frac{\partial }{\partial B}R_{k} & \approx \delta^{\frac{2}{\alpha }}\rho \left(K\right)\phi \left(P,K\right), \end{array}$$

In Figure 5, the gap between the simulation and the analysis results increases as the number of receive antennas increases. This is because the approximation in the SCVQ model is valid for small number of receive antennas. Thus, the gap of optimal number of feedback bits derived from the proposed SCVQ model also increases as the number of antennas increases. However, its behavior tracks that of the simulation results.

**Figure 6:**
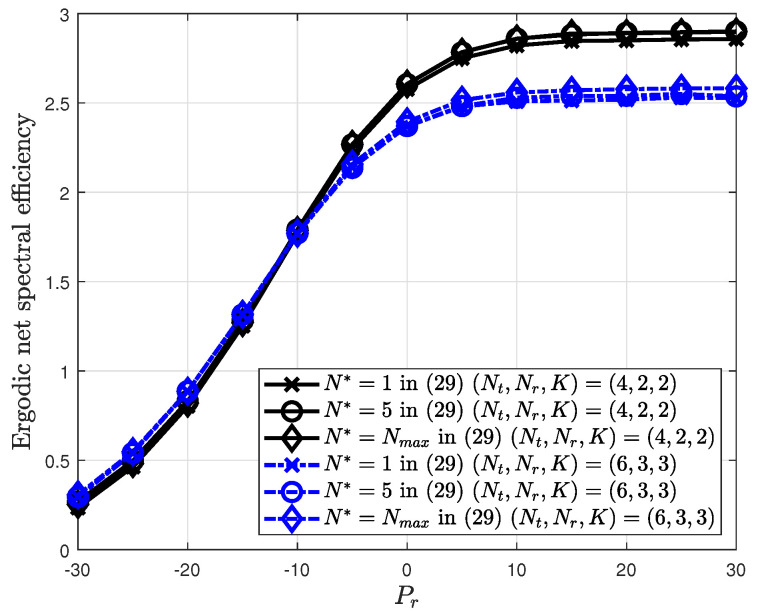
Ergodic net spectral efficiency according to number of interferers in the cellular networks.

Figure 6 shows the effectiveness of the calculation of the inter-cell interference.

**Figure 7:**
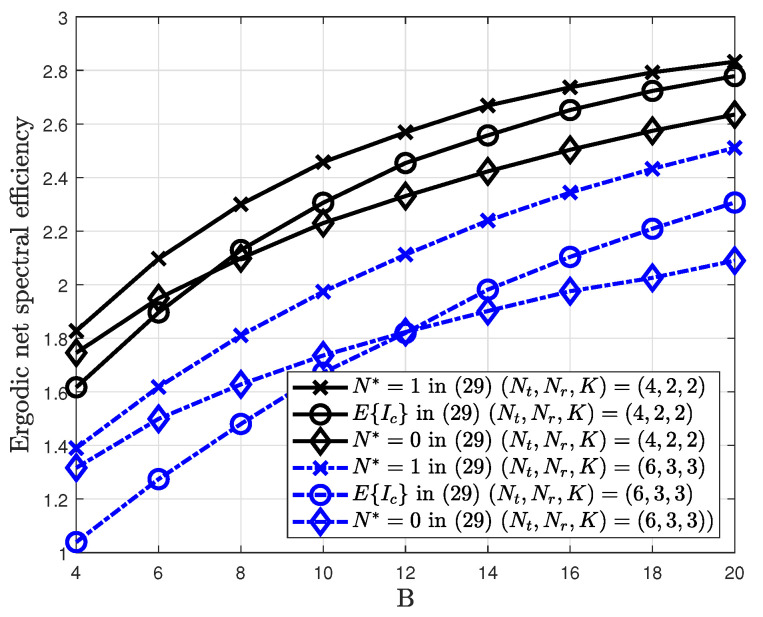
Ergodic net spectral efficiency for different values of the inter-cell interference.

For comparison, the expected value as $\frac{2K}{2-\alpha }$ in Equation (19) and zero value with $N^{*}=0$ are simulated. At lower number of feedback bits, the ergodic net spectral efficiency that ignores the inter-cell interference is better than that with the expectation of the inter-cell interference. This is because the intra-cell multiuser interference induced by a quantization error is more important than the inter-cell interference in this region. As the number of feedback bits *B* increases, the intra-cell multiuser interference is reduced; thus, the inter-cell interference is dominant. The proposed method with $N^{*}=1$ considers this interference value in the SINR calculation and shows the best performance compared to two methods. Figure 7 shows the ergodic net spectral efficiency for different numbers of adjacent cells $N^{*}$.

It is observed that the difference in the ergodic net spectral efficiency is small as the number of adjacent cells $N^{*}$ increases. From this observation, $N^{*}=1$ is used which corresponds to the nearest interfering cell from the user.

**Figure 8:**
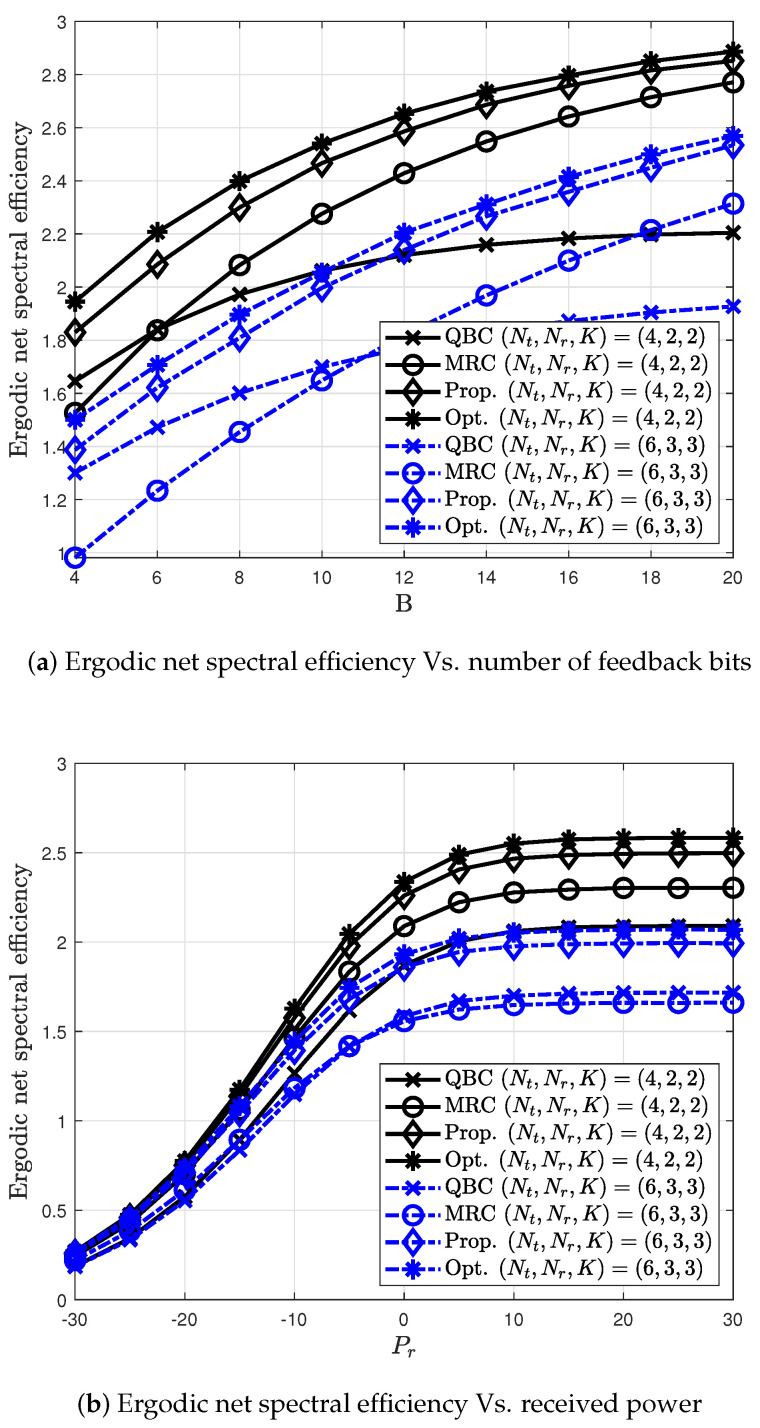
Ergodic net spectral efficiency of the proposed antenna combining.

Figure 8 shows the ergodic net spectral efficiency for the proposed antennas combining selection. For comparison, the optimal solution of Equation (23) is simulated. It is obtained on the assumption that the receiver has perfect knowledge of CSI for other cells, which is impractical in realistic networks. In Figure 8a, the proposed method outperforms than the existing methods as the proposed method chooses the best antenna combining for a given instantaneous channel.

For a large number of feedback bits, its multiplexing gain eventually follows that of the MRC method. Thus, the proposed method can inherit spatial multiplexing gain as the number of feedback bits increases. In Figure 8b, the proposed antenna combining selection is simulated at B=10. The gap in the ergodic net spectral efficiency between the proposed method and the existing methods is higher as the $P_{r}$ increases and eventually is saturated. In addition, it is observed that the gap between the proposed solution and the optimal solution is small.

## 6. Conclusions

In this paper, an antenna combining solution was investigated for interference limited MIMO cellular networks. From the comparison of the existing methods, it was found that the inter-cell interference limits the gain of the QBC. This degradation was analyzed by using the proposed SCVQ model. It was found that the effective channel gain is reduced by the QBC. To improve the degradation in effective channel gain, an optimization problem was proposed that selects the best antenna combining by comparing the expectation of the spectral efficiency. To obtain the expected value, the beamforming vectors of other cells were averaged. Then, by exhaustive search in the discrete set, the optimal solution of the proposed problem was obtained. The analysis results based on the proposed SCVQ model were verified with the simulation results. In addition, it was shown that the proposed selective method outperforms the conventional antenna combining methods.

## Appendix A. Proof of Lemma 1

Each user chooses the codebook index that minimizes the quantization error. When the quantization error between user *k* and each codebook index *i* is $\sin^{2}\theta_{k,i}$, the quantization error of user *k* is defined as

(A1) $$\begin{array}{cc} \sin^{2}\theta_{k} & =\min_{i=1,\ldots ,2^{B}}\;\sin^{2}\theta_{k,i},\\ \hat{k} & =\underset{i=1,\ldots ,2^{B}}{\mathrm{argmin}}\;\sin^{2}\theta_{k,i}, \end{array}$$

where $\sin^{2}\theta_{k}$ denotes the quantization error. Then, its CDF can be calculated as

(A2) $$\begin{array}{cc} F_{\sin^{2}\theta_{k}}\left(x\right) & =P\left(\sin^{2}\theta_{k}\leq x\right)=P\left(\sin^{2}\theta_{k,i}\leq x|\hat{k}\in R_{i}\right)=\frac{P(\sin^{2}\theta_{k,i}\leq x|\sin^{2}\theta_{k,i}\leq \delta )}{P(\sin^{2}\theta_{k,i}\leq \delta )}. \end{array}$$

The distribution of $\sin^{2}\theta_{k,i}$ follows $\beta (N_{t}-N_{r},N_{r})$ in [18] which is given by

(A3) $$\begin{array}{cc} f_{\sin^{2}\theta_{k,i}}\left(x\right) & =\frac{1}{B(N_{t},N_{r})}x^{N_{t}-N_{r}-1}{\left(1-x\right)}^{N_{r}-1}. \end{array}$$

Thus, the denominator of Equation (A2) is calculated as

(A4) $$\begin{array}{cc} P(\sin^{2}\theta_{k,i}\leq x) & =\int_{0}^{x}\frac{1}{B(N_{t},N_{r})}z^{N_{t}-N_{r}-1}{\left(1-z\right)}^{N_{r}-1}dz\\ \; & \overset{\left(a\right)}{\approx }\int_{0}^{x}\frac{1}{B(N_{t},N_{r})}z^{N_{t}-N_{r}-1}dz=\frac{1}{B(N_{t},N_{r})}\frac{x^{N_{t}-N_{r}}}{N_{t}-N_{r}}. \end{array}$$

In (a), (1−z) term is dropped by assuming that *z* is sufficiently small for a large *B*.

By applying Equation (A4) to Equation (A2), it is given by

(A5) $$\begin{array}{cc} F_{\sin^{2}\theta_{k}}\left(x\right)\; & \approx \left\{\begin{array}{cc} \frac{x^{N_{t}-N_{r}}}{\delta^{N_{t}-N_{r}}} & ,0\leq x\leq \delta ,\\ 1 & ,\delta \leq x, \end{array}\right. \end{array}$$

When $\delta =2^{-\frac{B}{N_{t}-N_{r}}}$ is fixed to give $P\left\{R_{i}\right\}=2^{-B}$, Equation (A5) is re-expressed as

(A6) $$\begin{array}{cc} F_{\sin^{2}\theta_{k}}\left(x\right) & \approx \left\{\begin{array}{cc} 2^{B}x^{N_{t}-N_{r}} & ,0\leq x\leq \delta ,\\ 1 & ,\delta \leq x, \end{array}\right. \end{array}$$

This completes the proof.
